# Optimization of phenolics and flavonoids extraction conditions of *Curcuma Zedoaria* leaves using response surface methodology

**DOI:** 10.1186/s13065-017-0324-y

**Published:** 2017-10-02

**Authors:** Nur Fauwizah Azahar, Siti Salwa Abd Gani, Nor Fadzillah Mohd Mokhtar

**Affiliations:** 1 Department of Agriculture Technology, Faculty of Agriculture, Universiti Putra Malaysia (UPM), 43400 Serdang, Selangor Malaysia; 2 Halal Products Research Institute, Universiti Putra Malaysia (UPM), 43400 Serdang, Selangor Malaysia; 3 Institute for Mathematical Research (INSPEM), Universiti Putra Malaysia (UPM), 43400 Serdang, Selangor Malaysia

**Keywords:** *Curcuma zedoaria*, Antioxidant activity, Response surface methodology, Phenolic, Flavonoids

## Abstract

This study focused on maximizing the extraction yield of total phenolics and flavonoids from *Curcuma Zedoaria* leaves as a function of time (80–120 min), temperature (60–80 °C) and ethanol concentration (70–90 v/v%). The data were subjected to response surface methodology (RSM) and the results showed that the polynomial equations for all models were significant, did not show lack of fit, and presented adjusted determination coefficients (R^2^) above 99%, proving their suitability for prediction purposes. Using desirability function, the optimum operating conditions to attain a higher extraction of phenolics and flavonoids was found to be 75 °C, 92 min of extraction time and 90:10 of ethanol concentration ratios. Under these optimal conditions, the experimental values for total phenolics and flavonoids of *Curcuma zedoaria* leaves were 125.75 ± 0.17 mg of gallic acid equivalents and 6.12 ± 0.23 mg quercetin/g of extract, which closely agreed with the predicted values. Besides, in this study, the leaves from *Curcuma zedoaria* could be considered to have the strong antioxidative ability and can be used in various cosmeceuticals or medicinal applications.

## Background

Plants are a substantial source of natural antioxidants. Active compounds present in natural antioxidants such as phenolic, carotenoids, flavonoids, folic acid, benzoic acid, and tocopherol are secondary metabolites of the plants which can provide various potential treatment and prevention of cancer, cardiovascular diseases, neurodegenerative diseases and etc. [1, 2].

Phenolics or polyphenols, including flavonoids, have received greater attention because they are often identified as biological response modifiers and have various functions such as metal chelators and free radical terminators [3, 4]. The bioactive compounds present in these compounds provide a variety of physiological functions, for instance, antimicrobial, antiallergenic, anti-inflammatory, and antimutagenic effects [5]. Moreover, it has been reported that the active compounds found in phenolic acids (caffeic, chlorogenic acid, benzoic acid) and flavonoids (catechin, quercetin, rutin) are potent antioxidants because they have all the right structural features for free radical scavenging activity [6, 7].

*Curcuma zedoaria* (Christm.) Roscoe. from Zingiberaceae family is popularly known as white turmeric, zedoaria or gajutsu [8]. This medicinal herb is largely found in East-Asian countries including Malaysia, Indonesia, China, India, Japan, Vietnam and Bangladesh [9]. Traditionally, zedoaria is hugely consumed as a spice, a flavoring agent, a tonic, a treatment for menstrual disorders, vomiting, toothache and it is also made into perfume [10, 11]. A study done by Angel et al. [12] reveals that zedoaria plants have a certain camphoraceous aroma and enormous functional active compounds such as essential oils, phenolics, and flavonoids which are strong components of anti-oxidant agent [12]. Meanwhile, Srivastava et al. [13] reported that *Curcuma zedoaria* is closely related to *Curcuma longa*. Therefore, the correlative isolated active compounds found in zedoaria such as curcumin, demethoxycurcumin and bisdemethoxycurcumin could be effectively used as antioxidant and anti-inflammatory, similar to *Curcuma longa* which is popularly used as antioxidant, antiulcer, anti-inflammatory, etc. Moreover, in vivo studies reported that the rhizomes of the plant possess potent antioxidant activity which exhibited higher radical scavenging activity [14].

The extraction of antioxidant compounds is a crucial process to determine the quantity and type of bioactive compounds, each with different therapeutic properties that will be extracted out. According to Aybastier et al. [15] many factors contribute to the efficiency of extractions such as the type of solvent, the concentration of solvent, temperature, time, pH and solid–liquid ratios. Response surface methodology (RSM) is a powerful mathematical technique being widely used in many industries for technological operations to optimize the experimental conditions. RSM is also useful to maximize or minimize various independent variables as it evaluates the effects of multiple factors and their respective interactions on one or more response variables simultaneously. Besides, RSM not only serves as a visual aid to have a clearer picture about the effects of various factors on extraction but also helps to locate the region where the extraction is optimized.

Therefore, the optimum extraction conditions (time, temperature and solvent ratio) to obtain the highest amount of phenolic and flavonoid compounds from *Curcuma zedoaria* leaves was identified using RSM technique. Despite numerous studies on rhizomes of zedoary which investigated its antioxidant activity, the leaves of the plant literally have not gained enough recognition and study to the best of our knowledge. In addition, Chanda and Nagani [16] reported that leaves, in general, are selected for the evaluation of total antioxidants activity due to high content of bioactive compounds.

## Results and discussion

### Fitting the response surface models

A full factorial, central composite design (CCD) was used to identify the relationship between the response functions and process variables as well as to find out the conditions that optimized the extraction process. The experimental design and corresponding three response variables are presented in Table 1. In the present study, according to the sequential model sum of squares, the highest order polynomials were utilized to select the models where the additional coefficients estimates were significant and the models are not aliased. Hence, for all three independent variables and responses, a quadratic polynomial model was selected and fitted well as suggested by the software.

**Table 1:** The experimental data obtained for the three responses based on the CCD matrix

Run no	Type	Temperature (*X*_*1*_)	Time (*X*_*2*_)	Solvent ratio (*X*_*3*_)	Phenolic content mg/g GAE	Flavonoid content mg QE/g extract
1	Fact	80.0	80.0	90.0	131.96	6.18
2	Fact	60.0	120.0	70.0	116.76	6.00
3	Center	70.0	100.0	80.0	122.20	6.24
4	Fact	80.0	120.0	70.0	122.90	6.07
5	Axial	53.18	100.0	80.0	116.14	6.09
6	Center	70.0	100.0	80.0	122.64	6.23
7	Axial	70.0	66.36	80.0	115.32	6.31
8	Axial	86.82	100.0	80.0	135.77	6.05
9	Axial	70.0	100.0	96.82	119.17	6.35
10	Fact	80.0	80.0	70.0	121.80	6.21
11	Axial	70.0	100.0	63.18	105.10	6.06
12	Fact	80.0	120.0	90.0	122.43	6.22
13	Fact	60.0	80.0	70.0	98.76	6.08
14	Fact	60.0	80.0	90.0	115.83	6.33
15	Center	70.0	100.0	80.0	122.32	6.23
16	Center	70.0	100.0	80.0	122.24	6.21
17	Fact	60.0	120.0	90.0	122.30	6.38
18	Axial	70.0	133.64	80.0	122.27	6.26
19	Center	70.0	100.0	80.0	122.55	6.22
20	Center	70.0	100.0	80.0	122.25	6.23

The final empirical regression model of their relationship between responses and the three tested variables for phenolic and flavonoid contents could be expressed by the following quadratic polynomial equation [Eqs. (1–2)]:

1 $$\begin{aligned} Phenolic \, content &= 122.36 + 5.74X_{1} + 2.03X_{2} + 4.10X_{3} \\ &\quad - 4.11X_{1} X_{2} - 1.62X_{1} X_{3} -2.77X_{2} X_{3} \\& \quad + 1.34X_{1}^{2} - 1.19X_{2}^{2} - 3.55X_{3}^{2} \end{aligned}$$

2 $$\begin{aligned} Flavonoid \, content &= 6.23 - 0.013X_{1} - 0.016X_{2} + 0.091X_{3} \\ & \quad - 0.08X_{1} X_{2} - 0.064X_{1} X_{3} + 0.039X_{2} X_{3}\\ &\quad \times 0.05X_{1}^{2} + 0.021X_{2}^{2} - 0.070X_{3}^{2} \end{aligned}$$

where *X*_*1*_ is the temperature, *X*_*2*_ is the time and *X*_*3*_ is the ethanol concentration ratio. A negative sign in each equation represents an antagonistic effect of the variables and a positive sign represents a synergistic effect of the variables.

The RSM model coefficients were validated by analysis of variance (ANOVA) of the response variables for the quadratic polynomial model summarized in Table 2. The ANOVA results were calculated based on 95% confidence intervals and this analysis was crucial to determine the best fitted quadratic model for three independent variables. A regression model was evaluated by using *F* statistics and lack of fit test. Based on the results, it showed that the model is highly significant when the computed *F*-value is greater than the tabulated *F*-value and the probability value is low (*p* < *0.0001*) indicating that the individual terms in each response model were significant on the interaction effect.

**Table 2:** Analysis of variance (ANOVA) for the model

Sources	Sum of squares	Degree of freedom	Mean squares	*F*-*value*	*p*-*value*
Phenolic content (mg/g GAE)					
Model	1191.21	9	132.36	1662.76	< 0.0001
*X*_*1*_-temperature	450.69	1	450.69	5661.84	< 0.0001
*X*_*2*_-Time	56.30	1	56.30	707.27	< 0.0001
*X*_*3*_-solvent ratio	229.32	1	229.32	2880.92	< 0.0001
*X*_*1*_ *X*_*2*_	135.30	1	135.30	1699.75	< 0.0001
*X*_*1*_ *X*_*3*_	20.87	1	20.87	262.13	< 0.0001
*X*_*2*_ *X*_*3*_	61.38	1	61.38	771.14	< 0.0001
*X*_*1*_^*2*^	25.87	1	25.87	325.02	< 0.0001
*X*_*2*_^*2*^	20.46	1	20.46	257.06	< 0.0001
*X*_*3*_^*2*^	181.23	1	181.23	2276.79	< 0.0001
Residual	0.80	10	0.080		
Lack of fit	0.63	5	0.13	3.74	0.0870
Pure error	0.17	5	0.034		
Cor total	1192.01	19			
R^2^ = 0.9993 Adj. R^2^ = 0.9987 CV% = 0.24					
Flavonoid content (mg QE/g of extract)					
Model	0.22	9	0.024	229.66	< 0.0001
*X*_*1*_-temperature	0.002	1	0.002	21.86	0.0009
*X*_*2*_-Time	0.003	1	0.003	31.89	0.0002
*X*_*3*_-solvent ratio	0.11	1	0.11	1065.88	< 0.0001
*X*_*1*_ *X*_*2*_	0.0006	1	0.0006	5.82	0.0365
*X*_*1*_ *X*_*3*_	0.033	1	0.033	308.93	< 0.0001
*X*_*2*_ *X*_*3*_	0.012	1	0.012	114.14	< 0.0001
*X*_*1*_^*2*^	0.044	1	0.044	421.70	< 0.0001
*X*_*2*_^*2*^	0.0006	1	0.0006	57.66	< 0.0001
*X*_*3*_^*2*^	0.0008	1	0.0008	8.25	0.0166
Residual	0.001	10	0.0001		
Lack of fit	0.0005	5	0.0001	0.97	0.5115
Pure error	0.0005	5	0.0001		
Cor total	0.22	19			
R^2^ = 0.9952 Adj. R^2^ = 0.9909 CV% = 0.17					

The performance of the models was also checked by calculating the determination coefficients *R* ^2^, adjusted *R* ^2^, regression (*p value*), regression (*F*-*value*), lack of fit (*p*-*value*), coefficient variation (C.V%) and probability values related to the effect of the three independent variables. Based on the result, the coefficient of determination *R* ^2^ is defined as the ratio of the explained variation to the total variation in total phenolic and total flavonoid contents were *R* ^2^ = 0.9993 and *R* ^2^ = 0.9952 respectively showing a good fit model. The closer *R* ^2^ value to unity, the better and significant empirical model fits the actual data. Furthermore, the calculated adjusted *R* ^2^ values for studied responses variables were higher than 0.80, hence there is a close agreement between the experimental results and the theoretical values predicted by the proposed models. The coefficients of variations (C.V) for total phenolic and flavonoid contents were 0.24 and 0.17 respectively, which indicates that a relatively lower value of CV showed a better reliability of the response model. It was observed that the lack of fit gave no indication of significance (*p* < *0.05*) for all the models tested, thus proving that the satisfactory fitness of the response surface model was within the chosen range and significant (*p* < *0.05*) to the factors effect.

Based on analysis of ANOVA, any terms from quadratic polynomial coefficients model, large *F*-*values* and a small *p*-*values* indicated a more significant effect on the respective response variables. The 3-D surface plots of the fitted polynomial regression equations were generated by the software to better visualize the interaction effect of independent variables on responses.

### Response surface analysis

Temperature, time and ethanol concentration are the main factors that affect the extraction condition of the maximum total phenolics and flavonoids content. This section discusses how these conditions work on natural antioxidants extraction. Three-dimensional model graphs were plotted as shown in the respective figures. The response surface plots of the model were done by varying two variables, within experimental range under investigation and holding the other variables at its central level (0 levels).

### Effects of process variables on the total phenolics content (TP)

**Fig. 1:**
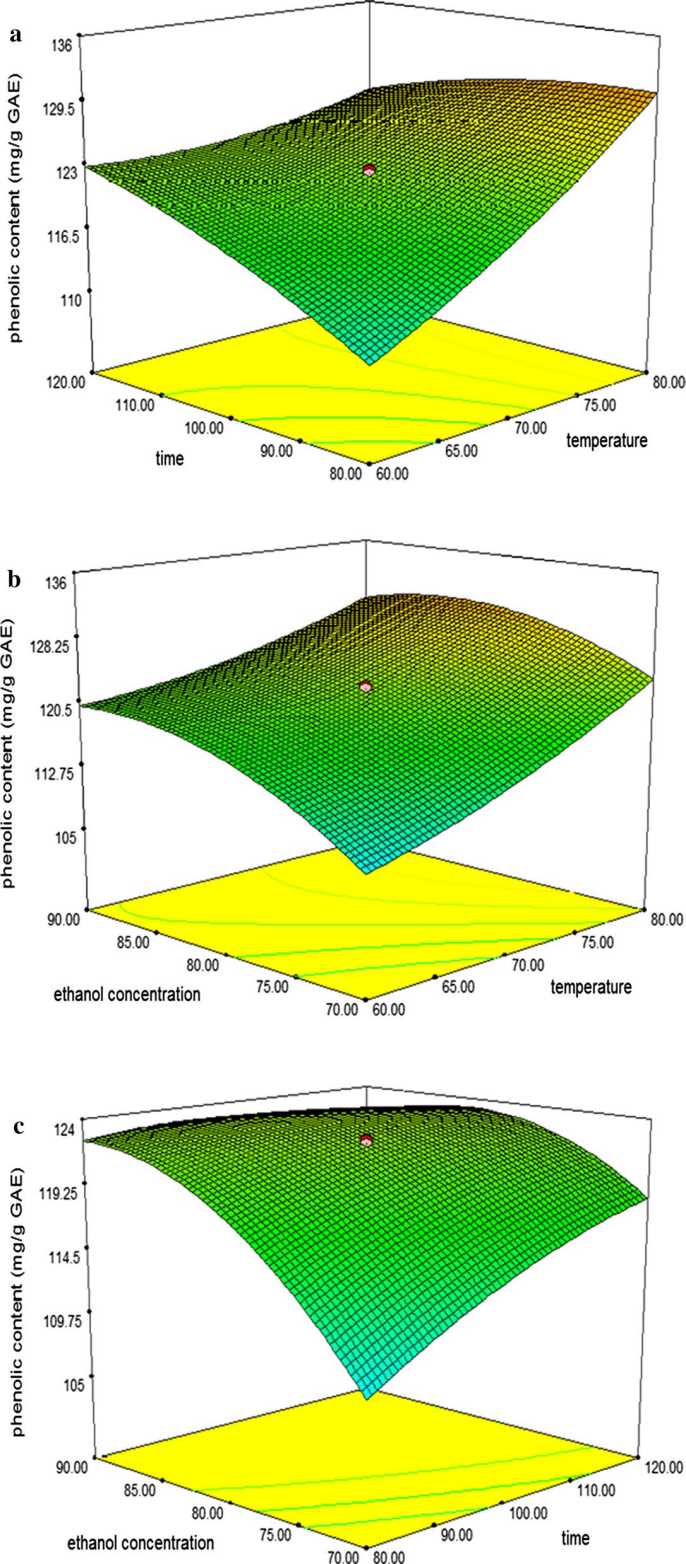
Response surface plots for the effects of temperature, time and ethanol concentration on total phenolic contents of *Curcuma zedoaria* leaves extracts. **a** Temperature versus time. **b** Ethanol concentration versus temperature. **c** Time versus ethanol concentration

The amount of extracted phenolics content from *Curcuma zedoaria* leaves ranged from 98 to 135 sample extract, measured as gallic acid equivalent (GAE). The value of mean recorded was 120.04 mg/g GAE of total leaves extracts. The highest TP content was reported at experimental run no. 8 while the lowest TP content was observed at experimental run no. 13. The ANOVA showed the model *F* value of 1662.76 with probability (p < 0.0001) which implies that the model is significant and there is only 0.01% chances that this large *F* value could occur due to noise. Phenolic content was significantly influenced at (p < 0.05) by all three linear (*X*_*1*_ *, X*_*2*_ *, X*_*3*_), interaction parameters (*X*_*1*_ *X*_*2*_ *, X*_*1*_ *X*_*3*_ *, X*_*2*_ *X*_*3*_) and quadratic parameters (*X*_*1*_^*2*^ *, X*_*2*_^*2*^ *, X*_*3*_^*2*^) (Table 2). The effect of their variables and their interaction on the responses can be seen in Fig. 1a–c.

The surface plot in Fig. 1a demonstrates the function of temperature (*X*_*1*_) versus time (*X*_*2*_) effect on total phenolic contents at fixed ethanol concentration (80:20). It was observed that increasing the extraction temperature and time resulted in higher phenolic content in *Curcuma zedoaria* leaves. The maximum amount of phenolics can be achieved at the highest temperature of 75–80 °C at the shortest extraction time of 80–100 min. Nevertheless, when the temperature was kept at the highest level of 80 °C with longer extraction time at 120 min, they did not show any significant improvement in TP extraction as the value continuously dropped. This agreed with the working high temperature employed in this study which required short periods of time to avoid the degradation of the phenolic compounds. At short periods of time, the temperature enhanced the extraction process but for relatively long periods, the effect is inverted and the phenolic compounds are threatened by oxidation or degradation [17]. Moreover, according to Vajić et al. [18] prolonged time of extraction enhances phenolic solubility due to Fick’s second law of diffusion which predicts that equilibrium of extraction will be achieved after a certain time. These results are similar to a study reported by of Rajha et al. [19] which showed the total phenolics from grape byproducts increased with the increment of temperature and reduction of time.

Figure 1b depicts the effects of temperature (*X*_*1*_) versus ethanol concentration (*X*_*3*_) at constant extraction time 100 min. The surface plot reveals that the maximum phenolic content can be achieved at highest ethanol concentrations (90:10) as compared with low ethanol concentrations (70:30) at fixed extraction temperature. The higher phenolic content could be explained by the natural polarity of the solvents used [20]. Ethanol and water were used in this study because they are safer to handle as compared to other organic solvents and more importantly, they are acceptable for human consumption. Samuagam et al. [21] stated that a suitable solvent ratio is able to improve the efficiency of extraction. The surface plots also reveal that by increasing the extraction temperature to higher levels, the amounts of phenolic gradually dropped and this might be explained by the fact that the final equilibrium between the solvent concentrations in the plant matrix and the temperature will be achieved after a certain concentration level [22]. This phenomenon is similar to a phenolic study from lettuce by-products which can be explained by the use of higher temperature and adequate solvent concentrations which may cause softening of plant tissue, resulting in enhanced diffusion rate and increase in the production of phenolic compounds. However, after a certain level, it will subsequently decline and remain constant as the extraction has completed and they have achieved their equilibrium state [23]. Therefore, the maximum total phenolic content in *Curcuma zedoaria* leaves can be obtained with optimum ethanol concentration and an extraction temperature of approximately 80–85 v/v% and 75–80 °C respectively.

The response surface plot as a function of time (*X*_*2*_) versus ethanol concentration (*X*_*3*_) at constant temperature 70 °C is presented in Fig. 1c. The surface plots revealed that the higher TP contents can be obtained when conducted at increasing ethanol concentration at fixed extraction time. Based on the result at constant extraction time of 120 min, 90% of ethanol concentrations yielded the most TP as compared with 70% ethanol concentrations. However, longer extraction time degrade the phenolic activity in *Curcuma zedoaria* leaves. Therefore the optimum extraction of phenolic can be obtained when conducted at a range of 80–90 v/v% and 100 min of ethanol concentrations and extraction time respectively. Beyond this optimal, the TP content declined. These overall results of phenolic content indicate a similar trend as observed in the phenolic content of tea (*camellia sinensis* L.) fruit peel by Xu et al. [24] where the TP contents increased with increasing the independent variables ethanol concentration and processing time until a maximum amount of phenolic was reached, thereafter, the amount subsequently declined rapidly as reaction has completed.

### Effects of process variables on the total flavonoids content (TF)

**Fig. 2:**
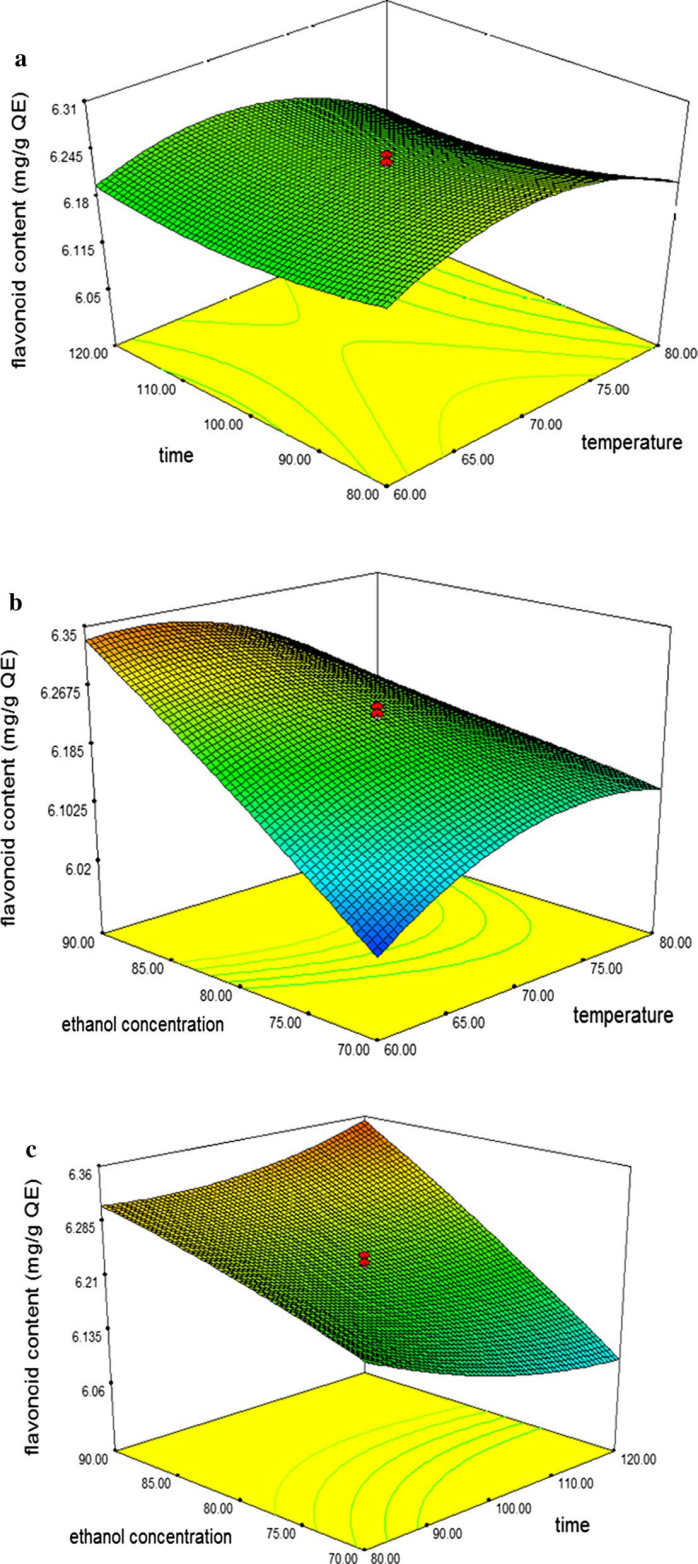
Response surface plots for the effects of temperature, time and ethanol concentration on total flavonoid content of *Curcuma zedoaria* leaves extracts. **a** Temperature versus time. **b** Ethanol concentration versus temperature. **c** Time versus ethanol concentration

The mean experimental data showing the total flavonoid content from *Curcuma zedoaria* leaves at various extraction conditions was 6.20 mg QE/g of extract in the total range of 6.00–6.38 mg QE/g of extract. The highest content of TF was observed at experimental run no. 17 meanwhile the lowest yield of TF was observed in experimental run no 2. The ANOVA showed the model *F* value of 229.66 with probability (p < 0.0001) which implies that the model is significant and there is only 0.01% chances that this large *F* value could occur due to noise. Flavonoids content was significantly influenced at (p < 0.05) by all three linear (*X*_*1*_ *, X*_*2*_ *, X*_*3*_), interaction parameters (*X*_*1*_ *X*_*2*_ *, X*_*1*_ *X*_*3*_ *, X*_*2*_ *X*_*3*_) and quadratic parameters (*X*_*1*_^*2*^ *, X*_*2*_^*2*^ *, X*_*3*_^*2*^) (Table 2). The effect of their variables and their interaction on the responses can be seen in Fig. 2a–c.

The 3D shows the response surface plot as a function of temperature (*X*_*1*_) versus time (*X*_*2*_) at fixed extraction ethanol concentration (80:20) as shown in Fig. 2a. Response surface plot showed that extraction temperature exhibited a weaker effect whereas extraction time represented a relatively significant effect on the flavonoids yield. An increase in the yield of flavonoid could be significantly achieved with the increase of extraction time, at any level of extraction temperature. Therefore, the optimum amount of flavonoid was achieved in this study at 65–70 °C and 90–100 min of extraction time. However, the results of the present research for time and temperature were different compared with other studies [4, 19]. This difference could be the due to differences in the type of material, considering some plants may synthesize and accumulate the different amount of secondary metabolites (flavonoids) and also the optimization extractions range used in the study.

The 3D surface plots in Fig. 2b shows the interaction between extraction temperature (*X*_*1*_) and ethanol concentration (*X*_*3*_) at the fixed 100 min. Statistical analysis reveals that the most significant with *p* < 0.0001 in TF was ethanol concentration. According to Bazykina et al. [25] flavonoids and their glycosides are thought to be efficiently extracted from plant materials by ethanol solvent. It was observed that the value of TF increased when ethanol concentration was increased from 70 to 90 v/v% at fixed 60 °C extraction temperature. In contrast, increasing the extraction temperature at highest ethanol concentrations resulted to decreased, TF values. This phenomenon can be explained by the higher movement of the particles which causes plant tissue to rupture and hence allowing higher solubility of solvent until it starts to degrade to a lower value as it had achieved the stable state [26]. The results obtained for flavonoids are in agreement with the previous report from *Cryptotaenia japonica hassk* by Lu et al. [27] where the flavonoid content increased when the temperature of extraction increased to below 70 °C and exhibited a decreasing trend above the optimum level of temperature. Thus, as mentioned earlier the optimum extraction temperature for maximum flavonoid content was at 65–70 °C with 85–90 v/v% ethanol concentrations.

Figure 2c illustrates the response surface plot between the extraction time (*X*_*2*_) and ethanol concentration (*X*_*3*_) at constant extraction temperature (70 °C). The response surface plots demonstrated that the value of TF obtained in *Curcuma zedoaria* leaves mainly depended upon ethanol concentrations. An increase in ethanol concentration promoted the breakdown of the cell membrane that enhanced the permeability of the solvent into a solid matrix. In this study, highest flavonoids content can be achieved when conducted at highest ethanol to water ratio (90:10) as compared with (70:30) with increasing extraction time. A great increase in the yield also resulted when extraction time was increased in the range of 80–120 min. However, the time curve started to level off at 100 min, which indicated that 100 min were required to achieve maximum flavonoids activity.

### Optimization of extracting parameters and validation of the model

In this study, the aim was to find the conditions which gave the maximum yield of total phenolic and flavonoids content. The final result for the simultaneous optimization using the desirability function approach suggested that the optimal ethanolic extraction conditions for *Curcuma zedoaria* leaves extract were at 75 °C with 92 min and 90:10 of ethanol concentration to achieve the best combination for highest total phenolic and flavonoids content. These optimum extraction conditions were evaluated by considering the simultaneous response surface and contour plot from the interaction between the independent variables and responses of interest. In order to verify the optimum conditions, the *Curcuma zedoaria* leaves were subjected using the optimal conditions above and the results were statistically compared to the predicted values given by the design expert 7.0.0 software of the response surface methodological (RSM) model. Based on the results, the predicted values of responses were found to be quite comparable with experimental values at 95% confidence level in Table 3.

**Table 3:** Comparison between the predicted and experimental values for antioxidants from extracts of *Curcuma zedoaria* leaves

Condition	Response values	
	Phenolic content mg/g GAE	Flavonoid content mg/g QE
Predicted	126.25	6.24
Experimental	125.75 ± 0.17	6.12 ± 0.23

## Conclusions

Response surface methodology (RSM) and a design called central composite design (CCD) were successfully developed to determine the optimum process parameters and the second order polynomial models for predicting responses were obtained. The best combination of extraction temperature, time and ethanol concentrations were found to be 75 °C with 92 min and 90:10 ethanol to water ratio which rendered a mean phenolic content of 125.75 ± 0.17 mg/g GAE and 6.12 ± 0.23 mg/g QE of total flavonoid content from experimental run and thus indicated good antioxidant activities from the leaves of *Curcuma zedoaria*.

## Materials and methods

### Raw materials

*Curcuma zedoaria* leaves were collected from a local farmer in Kedah, Malaysia. The chemicals, sodium carbonate, aluminium chloride, ethanol was purchased from J. Kollin Chemicals, Germany. Folin-Ciocalteu’s phenol reagent, gallic acid and quercetin were purchased from Sigma-Aldrich (St. Louis, MO, USA). All other chemical reagents used in this study were of analytical grade class.

### Plant extraction

The air-dried leaves of *Curcuma zedoaria* plant were cut into pieces and ground into powder form using a mechanical blender. About 0.5 g of powdered leaves were exactly weighed into a 150 mL round bottomed flask and mixed with ethanol. The extraction process was performed using a reflux systems equipped with a temperature controller and digital timer. The extract was then filtered through normal filtration using Whatman filter paper and vacuum-dried in a rotary evaporator, at 40 °C until the excess solvent was completely removed.

### Experimental design

The optimization of the extraction conditions from the *Curcuma zedoaria* leaves was established by using response surface methodology (RSM). This powerful mathematical and statistical technique is useful for modeling and analysis of problems in which a response is influenced by several independent variables and the objective is to find the relationship between the factor and the response to optimize the conditions. A design expert software Version 7.0.0, (Stat ease Inc., Minneapolis, USA) was used in this study. The experimental plan was carried out based on three factor/five level design referred to as rotatable central composite design (CCD). The selection of CCD as the experimental design is because it is more precise for estimating factor effects [28]. Hence, the interaction effect between factors can be evaluated and optimized in the full factor space.

The design consisted of twenty experimental runs, including six replicates at the center points. The center points were utilized to define the experimental error and the reproducibility of the data. The independent variables in this study were extraction temperature (*X*_*1*_: 60–80 °C), time (*X*_*2*_: 80–120 min) and ethanol concentrations (*X*_*3*_: 70–90% v/v ethanol/water). The five levels of values for the independent variables were explicit of their coded and uncoded forms in Table 4. The value of independent variables was expressed in their coded values as −1, 0, +1 interval shows the low, center, and high level of each variable, respectively. The multiple regression analysis was performed on the data of response variables such as total phenols and flavonoid content obtained as affected by the extraction conditions and was fitted into a polynomial regression equation as shown in the following equations (Eq. 3);

3 $$Y = \beta_{o } + \mathop \sum \limits_{i = 1}^{k} \beta_{i } \, X_{i} + \mathop \sum \limits_{i = 1}^{k} \beta_{ii } \, X_{i}^{2} + \mathop \sum \limits_{i = 1}^{k} \mathop \sum \limits_{j i}^{k} \beta_{ij } \, X_{i} X_{j} + e$$

where *Y* represents the response variables to be modeled; *β*_*o*_ is a constant, *β*_i_,*β*_ii_ and *β*_ij_ are the linear, quadratic and cross-product coefficients, respectively. *X*_*i*_ and *X*_*j*_ are the levels of the independent variables. *k* is the number of variables and *e* is the random error of the model.

**Table 4:** Independent test variables and their coded and uncoded value used for CCD matrix

Variables	Units	Coded & uncoded level of variables				
		−α	−1	0	1	+α
Temperature, *X*_*1*_	°C	53	60	70	80	87
Time, *X*_*2*_	Min	66	80	100	120	133
Solvent ratio, *X*_*3*_ Ethanol:water	v/v%	63	70	80	90	97

### Determination of total phenolic content

The total phenolic compounds in *Curcuma zedoaria* leaves was developed using the method of Singleton and Rossi [29] with minor modifications. For each sample, 100 μL (1 mg/mL) of the sample extract was mixed with 50 μL Folin-Ciocalteu’s reagent (2 N) previously diluted with 7.9 mL distilled water. After 4 min, 1.5 mL of 7.5 w/v% sodium carbonate solution was added to the mixture and incubated in the dark room at room temperature for 2 h. The absorbance values of the sample were measured at 765 nm using a UV–VIS microplate reader. Standard of gallic acid with different concentrations (25–1000 μg/L) was prepared in this study to generate a standard calibration curve. The samples were calculated based on the standard calibration curve and were expressed as mg gallic acid equivalent (mg/g GAE).

### Determination total flavonoids content

The content of flavonoid in the studied leaves extract was determined using spectrophotometric method [30]. From each sample, 100 μL (1 mg/mL) were mixed with 2% AlCl_3_ and incubated for 15 min at room temperature. The absorbance was measured at λ = 406 nm. The same procedure was repeated for the standard solution of quercetin at different concentrations (25–250 μg/mL) and the calibration line was obtained. Based on the measured absorbance, the concentration of flavonoids was calculate (mg/mL) on the calibration line and the content of flavonoids in extracts was expressed in terms of quercetin equivalent, QE (mg of quercetin/g of extract).

### Statistical analysis and optimization

Best fitted model of response can be achieved by highlighting these statistical parameters including the adjusted multiple correlation coefficients (*adjusted R* ^*2*^), multiple correlation coefficients (*R* ^2^), coefficient variation (C.V%), lack of fit, regression *F*-*value* and regression *p*-*value* by using analysis of variance (ANOVA). This statistical approach was used to summarize the results obtained under all experimental conditions with a confidence interval of 95% set to test the significant effect of the factors and their interaction. The optimal extraction conditions were selected based on the condition of achieving the highest total phenolics and flavonoids content in *Curcuma zedoaria* leaves by using the desirability function approach in design expert software. The fitted polynomial equation was expressed in the form of three-dimensional surface plots in order to illustrate the relationship between responses and the experimental variables used.

### Verification of models

The optimal conditions for the extraction of the total phenolic and flavonoid content from *Curcuma zedoaria* leaves, in terms of extraction temperature, time and ethanol concentrations, were determined by comparing the actual experimental values with predicted value from the final response regression equations. Besides, a few random extraction conditions were prepared in order to validate the models. This action is of utmost importance to confirm the adequacy of the final reduced models.
